# BOSC 2019, the 20th annual Bioinformatics Open Source Conference

**DOI:** 10.12688/f1000research.21568.1

**Published:** 2019-12-20

**Authors:** Nomi L. Harris, Peter J.A. Cock, Christopher J. Fields, Bastian Greshake Tzovaras, Michael Heuer, Karsten Hokamp, Monica Munoz-Torres, Alexander Peltzer, Bastian Rieck, Heather Wiencko, Yo Yehudi

**Affiliations:** 1 Lawrence Berkeley National Laboratory, Berkeley, CA, 94720, USA; 2 Information and Computational Sciences, James Hutton Institute, Dundee, DD2 5DA, UK; 3 Carver Biotechnology Center, University of Illinois at Urbana-Champaign, Urbana, IL, 61801, USA; 4 Center for Research & Interdisciplinarity, Université de Paris, Paris, France; 5 University of California, Berkeley, Berkeley, CA, 94720, USA; 6 Smurfit Institute of Genetics, Trinity College Dublin, Dublin, Ireland; 7 Environmental and Molecular Toxicology, Oregon State University, Corvallis, OR, 97331, USA; 8 Quantitative Biology Center, University of Tübingen, Tübingen, 72074, Germany; 9 Machine Learning & Computational Biology Lab, ETH Zürich, Zürich, Switzerland; 10 Open Bioinformatics Foundation, Dublin, Ireland; 11 Department of Genetics, University of Cambridge, Cambridge, CB2 3EH, UK

**Keywords:** bioinformatics, open source, open science, conference

## Abstract

The Bioinformatics Open Source Conference is a volunteer-organized meeting that covers open source software development and open science in bioinformatics. Launched in 2000, BOSC has been held every year since. BOSC 2019, the 20th annual BOSC, took place as one of the Communities of Special Interest (COSIs) at the Intelligent Systems for Molecular Biology meeting (ISMB/ECCB 2019). The two-day meeting included a total of 46 talks and 55 posters, as well as eight Birds of a Feather interest groups. The keynote speaker was University of Cape Town professor Dr. Nicola Mulder, who spoke on “Building infrastructure for responsible open science in Africa”.

Immediately after BOSC 2019, about 50 people participated in the two-day CollaborationFest (CoFest for short), an open and free community-driven event at which participants work together to contribute to bioinformatics software, documentation, training materials, and use cases.

## Introduction

As Europe experienced a record-breaking heat wave, BOSC 2019 attendees stayed cool in the Basel Congress Center (and many took breaks by anchoring down the Rhine). This was the 20th annual Bioinformatics Open Source Conference, a meeting held as one of more than a dozen ‘Communities of Special Interest’ (COSIs) at ISMB, the flagship meeting of the International Society for Computational Biology. (In 2018, BOSC partnered with the Galaxy Community Conference in GCCBOSC2018.)

**Figure 1.**
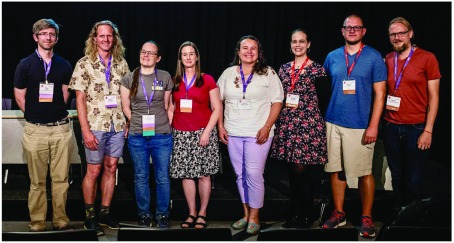
The BOSC 2019 Organizing Committee. From left to right: Peter Cock, Karsten Hokamp, Yo Yehudi, Nomi Harris, Monica Munoz-Torres, Heather Wiencko, Michael Heuer, Bastian Greshake Tzovaras; not shown: Chris Fields. Photo by Scott Edmunds. This figure is shared under the CC BY-SA 2.0 license.

BOSC is organized by the Open Bioinformatics Foundation (OBF), a non-profit, volunteer-run group that promotes open source software development and open science within the biological research community. The BOSC organizing committee ( Figure 1) works year-round to plan the annual meeting.

## Program

**Figure 2.**
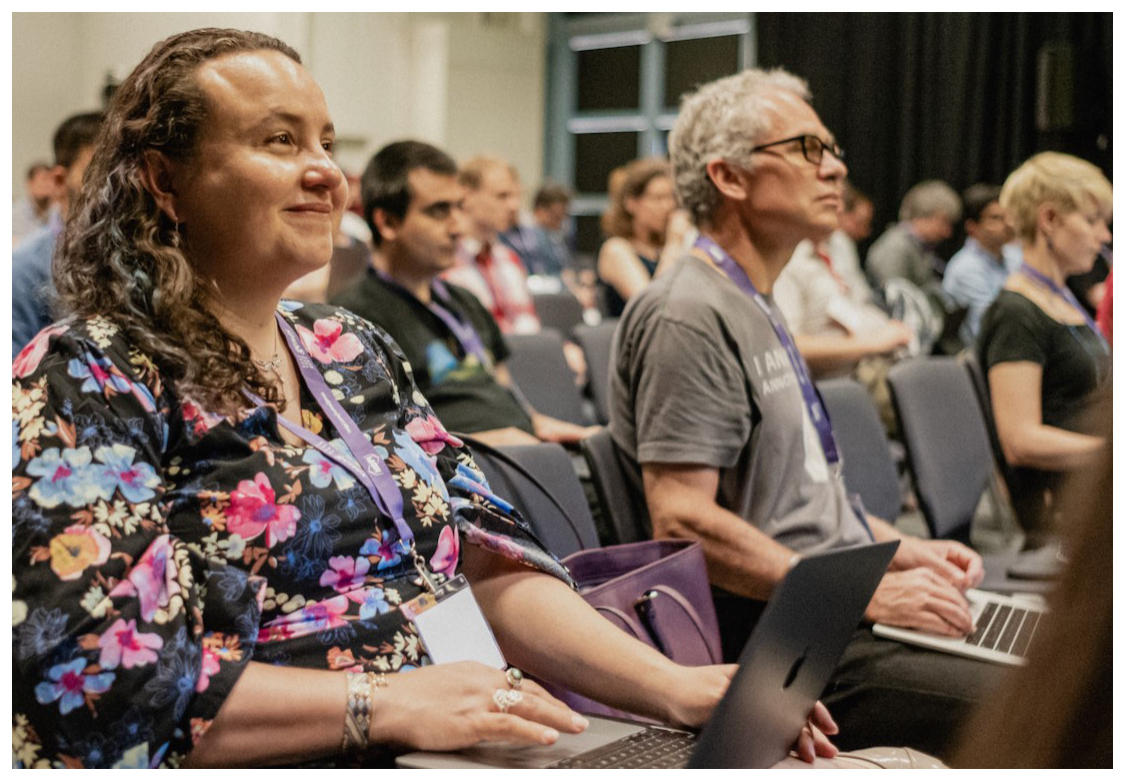
A rapt audience at BOSC 2019. Photo by Bastian Greshake Tzovaras. This figure is shared under the CC BY-SA 2.0 license.

**Figure 3.**
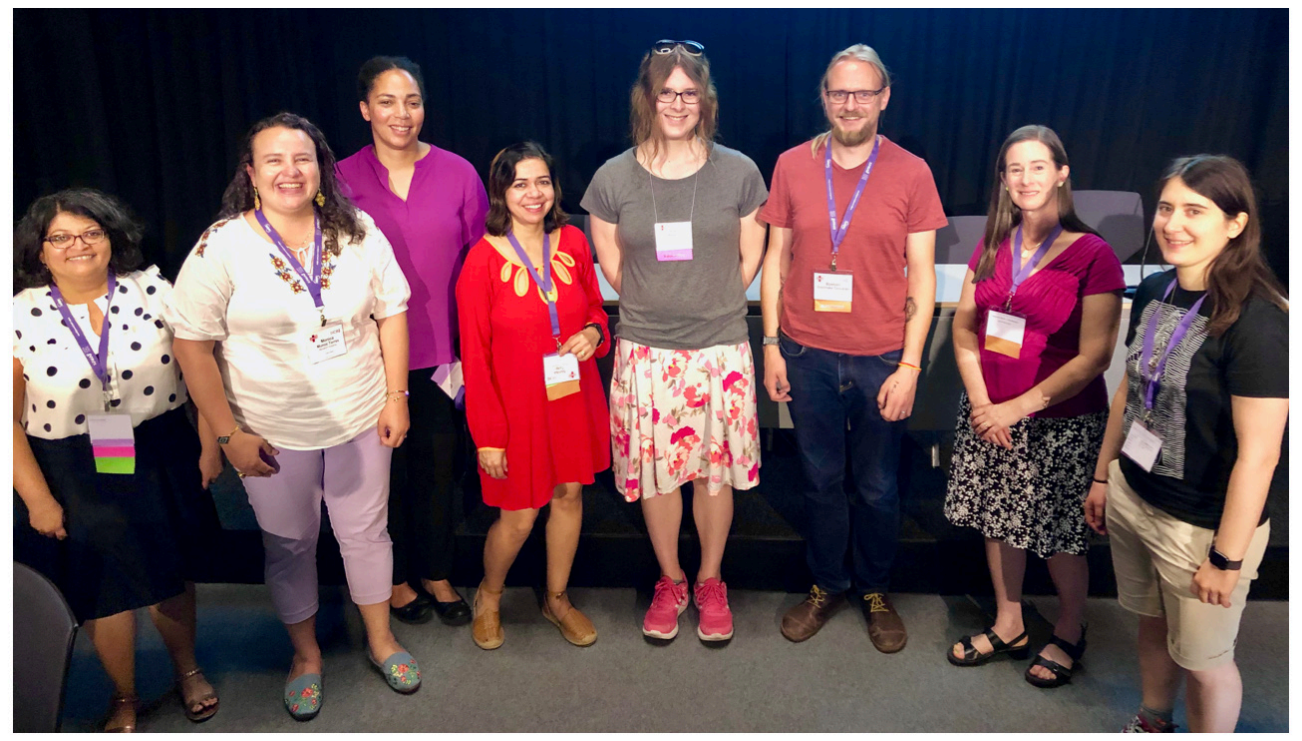
Some of the attendees at the “Welcome to BOSC” BoF. Photo by Scott Edmunds. This figure is shared under the CC BY-SA 2.0 license.

BOSC 2019 took place July 24–25, the last two days of the four-day ISMB/ECCB conference. The two-day meeting ( Figure 2) included a total of 46 talks and 55 posters, as well as eight self-organized Birds of a Feather interest groups ( Figure 3). During the meeting, attendees generated over 1500 tweets mentioning #BOSC2019 (you can find them in JSON format here).

The meeting opened with chair Nomi Harris noting that over its 20 years of history, BOSC has been held in 12 different countries, 6 US states and 2 Canadian provinces. BOSC co-chair Heather Wiencko followed these remarks with details about the Open Bioinformatics Foundation, BOSC’s parent organization, and Kai Blin discussed the OBF’s participation in Google Summer of Code.

The talks at BOSC were, as usual, organized into topic-based sessions, which included four recurring topic sessions:

- Open data
- Open science
- Workflows
- Late-breaking Lightning Talks

This year, BOSC added four new sessions to the program:

- Data crunching
- Data modeling and formats
- Containers
- Building Open Source Communities

Two of the new sessions, *Data crunching* and *Data modeling and formats*, discussed different aspects of working with data: representing it, storing it, analyzing it, and sharing it, all in the context of open source software and open data. The new *Containers* session discussed the use of containers such as Docker for enabling reproducible run-anywhere analyses. The popular *Workflows* session covered tools and metrics for CWL, the workflow language that was germinated during BOSC 2014, as well as other workflow systems. The *Building Open Source Communities* session (a new session that shares a familiar acronym with our conference) was devoted to efforts in open source and open science that go beyond software; for example, there were talks about training efforts, social networking in science, and ways to promote diversity and inclusivity in open source communities.

### Keynote

This year’s keynote speaker was University of Cape Town professor Nicola Mulder, who spoke on “Building infrastructure for responsible open science in Africa”. In her keynote, she highlighted how sharing data in Africa involves technical, ethical and social challenges, observing that “It’s really hard to convince people to share their data and their tools when they have such a history of being exploited.” Despite these obstacles, the H3ABioNet Consortium that Prof. Mulder leads is making progress in building a pan-African bioinformatics network offering a diverse set of services across the continent. Among other things, they (1) provide distributed training, with a recent iteration teaching 700 people across Africa simultaneously; (2) support an African Open Science Platform; and (3) run crowdsourcing challenges to design predictive models for Malaria.

### Birds of a Feather

Birds of a Feather (BoFs) are informal, self-organized meetups focused on specific topics. They are a great way to meet other like-minded community members and have an in-depth discussion on a topic of interest. BoFs, which can be proposed and run by any attendee, are always a popular part of BOSC. BOSC 2019 had eight BoFs:

- Welcome to BOSC ( Figure 3): This now-traditional BoF is designed to welcome both new and returning members, answer their questions, and gather feedback to help the organizers make future meetings even better.
- Common Workflow Language: Participants learned about using CWL to create and run data analysis workflows in a portable and interoperable manner, and shared their experiences with writing and running CWL.
- Reimagining the paper: This BoF offered an opportunity to discuss and critique tools designed to drive reproducible research.
  KNIME: Attendees learned about the open source KNIME Analytics Platform for reproducible interactive data analysis, and offered feedback on use cases and features.
- Variant Storage and Analysis: Participants in this BoF discussed challenges and solutions for ingesting and storing variant data.
- The Open Bioinformatics Foundation public board meeting: This is discussed in the next section.
- Cloud Capable Data and Tooling: This BoF focused on pain points and opportunities for working with large datasets in the cloud.
- Biopython and Biotite Collaboration: Participants in this BoF discussed how to bring together the best aspects of both of these Python-focused projects.

### OBF Board Meeting

**Figure 4.**
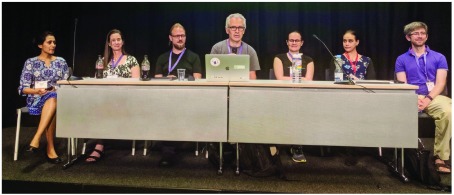
The OBF Board (including newly-elected member Malvika Sharan, far left) at the public Board meeting held during BOSC 2019. Photo by Michael Crusoe. This figure is shared under the CC BY-SA 2.0 license.

The OBF, an organization open to anyone who demonstrates interest in open source or open science in biology, holds Board meetings at least once a year that are open to the public. The July 2019 public Board meeting ( Figure 4) was held at ISMB/ECCB 2019, and was open to any conference attendees who wanted to participate, as well as to OBF members participating remotely. Public OBF Board meetings give OBF members an opportunity to vote on new Board member candidates and officers. At this meeting, Malvika Sharan was elected as a new Board member. Further topics of discussion included plans for the creation of an OBF-wide code of conduct that will cover new member projects and in-person events, potential changes to the OBF membership policies, and the community-assisted crafting of a mission statement for the OBF.

## CollaborationFest

**Figure 5.**
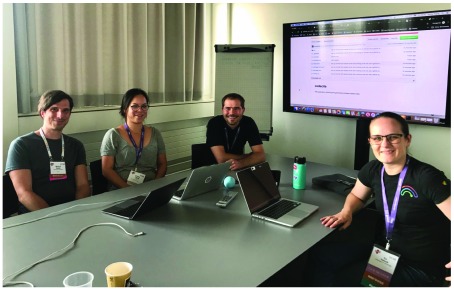
Some of the CodeCite team at CoFest 2019. This group worked on software citation tools that can be used to generate CFF (citation file format) based on existing metadata on GitHub or Zenodo. Photo by Sonika Tyagi. This figure is shared under the CC BY-SA 2.0 license.

The two days after BOSC, about 50 people participated in the OBF-run CollaborationFest (CoFest for short), an event at which participants work together to contribute to open source bioinformatics software, documentation, training materials, and use cases. Organized by Alexander Peltzer, Michael Heuer, Peter Cock, and Bastian Rieck, CoFest 2019 was held at DayOne, The Swiss Innovation Hub for Personalized Medicine in Basel ( Figure 5). Participants worked together to fix bugs, add new features, write tests, and improve documentation in new and ongoing projects including Biopython, Common Workflow Language (CWL), Nextflow, Cannoli, CodeCite, the Synthetic Sequence Data Project, and the OBF’s affiliated project policy.

## Plans for BOSC 2020

BOSC 2019 closed with an announcement: next year’s meeting will be held in collaboration with Galaxy’s Community Conference as the Bioinformatics Community Conference ( BCC2020), which will take place in Toronto, Canada, July 19–22, 2020. BCC2020 will include a training day, the main two-day conference, two CoFest days, and two additional optional CoFest Encore days.

### Consent for publication

All photos in this report are shared under a CC BY-SA 2.0 license. All photographers consented to sharing their photos. All identifiable subjects in the photos were contacted and they consented to have their photos published in this report.

